# Surgical Challenges During the COVID-19 Crisis: A Comparative Study of Inguinal Hernia Treatment in Romania

**DOI:** 10.3390/medicina60111825

**Published:** 2024-11-06

**Authors:** Catalin Vladut Ionut Feier, Calin Muntean, Vasile Gaborean, Razvan Constantin Vonica, Alaviana Monique Faur, Marius-Sorin Murariu, Sorin Olariu

**Affiliations:** 1 Abdominal Surgery and Phlebology Research Center, “Victor Babeş” University of Medicine and Pharmacy, 300041 Timisoara, Romania; catalin.feier@umft.ro (C.V.I.F.); murariu.marius@umft.ro (M.-S.M.); olariu.sorin@umft.ro (S.O.); 2 First Surgery Clinic, “Pius Brinzeu” Clinical Emergency Hospital, 300723 Timisoara, Romania; 3 Medical Informatics and Biostatistics, Department III-Functional Sciences, “Victor Babeş” University of Medicine and Pharmacy Timişoara, Eftimie Murgu Square No. 2, 300041 Timisoara, Romania; cmuntean@umft.ro; 4 Thoracic Surgery Research Center, “Victor Babeş” University of Medicine and Pharmacy Timişoara, Eftimie Murgu Square No. 2, 300041 Timisoara, Romania; 5 Department of Surgical Semiology, Faculty of Medicine, “Victor Babeş” University of Medicine and Pharmacy Timişoara, Eftimie Murgu Square No. 2, 300041 Timisoara, Romania; 6 Preclinical Department, Discipline of Physiology, Faculty of Medicine, “Lucian Blaga” University of Sibiu, 550169 Sibiu, Romania; razvanconstantin.vonica@ulbsibiu.ro; 7 Faculty of Medicine, “Victor Babeş” University of Medicine and Pharmacy Timişoara, 300041 Timisoara, Romania; alaviana.faur@student.umft.ro

**Keywords:** COVID-19 pandemic, inguinal hernia repair, elective surgery, hospitalization

## Abstract

*Background and Objectives*: The COVID-19 pandemic disrupted healthcare systems worldwide, leading to the postponement of elective surgeries, including inguinal hernia repair (IHR), as healthcare resources prioritized critical care. This study aims to evaluate the impact of the pandemic on the incidence and outcomes of IHR procedures. *Materials and Methods*: A retrospective review was conducted on 604 patients who underwent IHR over six years, spanning pre-pandemic, pandemic, and post-pandemic periods. Data on patient demographics, type of surgical procedure (elective or emergency), use of mesh, surgical duration, hospitalization period, and postoperative outcomes were analyzed across the three time frames. *Results*: Patient age remained consistent across the three periods, but a significant increase in female patients was observed during and after the pandemic (*p* < 0.001). Elective IHR surgeries significantly decreased during the pandemic (*p* < 0.001), paralleled by an increase in emergency cases (*p* = 0.004). In the post-pandemic period, elective surgeries rebounded, while emergency interventions declined (21.9% vs. 10.3%). Mesh repair usage increased notably in the post-pandemic phase (*p* < 0.001). Although surgeries took longer during the pandemic (*p* < 0.001), both total and postoperative hospital stays were reduced during and after the pandemic (*p* < 0.001). Minimal postoperative complications were reported throughout, with only one mortality during the pandemic. *Conclusions*: This study highlights the need for robust healthcare strategies to maintain elective surgical care during global crises, as delays in IHR may elevate risks for complications like hernia incarceration and strangulation.

## 1. Introduction

The COVID-19 pandemic caused significant disruptions in healthcare systems globally, with long-term consequences that are still not fully understood [1]. Romania ranked among the top 50 most affected countries worldwide, reporting over 150,000 COVID-19 cases per million inhabitants, and placed among the top 20 most impacted countries in Europe. The first wave of the pandemic in Romania led to a nationwide lockdown beginning in March 2020, with restrictions persisting until their final removal in March 2022 [2].

Elective procedures, including hernia repairs, were deprioritized as healthcare resources and personnel were reallocated to manage the influx of COVID-19 patients. This reallocation resulted in a significant reduction in elective surgeries globally, with estimates suggesting that over 28 million elective surgeries were canceled worldwide in 2020, including inguinal hernia repairs [3]. Surgical intervention for inguinal hernias, typically performed electively, are one of the most common surgical procedures [4,5]. If left untreated, inguinal hernias can lead to severe complications, such as incarceration or strangulation, potentially progressing to bowel necrosis and other life-threatening conditions [6].

Prior to the pandemic, elective surgical intervention was regarded as the preferred approach, given its lower risk profile compared to emergency surgeries. However, the pandemic necessitated the postponement of many elective procedures, including hernia repairs, to allocate critical care resources for COVID-19 patients. This postponement led to a surge in emergency cases, as patients whose elective surgeries were deferred experienced deteriorating conditions requiring urgent intervention [7,8]. The shift from elective to emergency surgeries placed substantial strain on healthcare systems and increased the risk of adverse outcomes, given the heightened complexity and urgency of delayed cases [9].

This six-year retrospective study, spanning from 2018 to 2024, aims to evaluate the impact of the COVID-19 pandemic on inguinal hernia repair surgeries in Romania, focusing on the procedure’s largely elective nature. By comparing clinical outcomes across pre-pandemic, pandemic, and post-pandemic periods, this study seeks to provide critical insights into how the pandemic disrupted elective surgical care.

## 2. Materials and Methods

This retrospective study investigates patients who received open surgical interventions for inguinal hernias at the First Surgery Clinic of the “Pius Brinzeu” Clinical Emergency Hospital in Timişoara. A total of 604 patients, treated between 26 February 2018 and 25 February 2024, were included in the analysis.

To assess the influence of the COVID-19 pandemic on the management of these patients, the study period was segmented into three distinct phases:

- Pre-pandemic group: 26 February 2018 to 25 February 2020.
- Pandemic group: 26 February 2020 to 25 February 2022.
- Post-pandemic group: 26 February 2022 to 25 February 2024.

The starting date of 26 February was specifically chosen because it coincides with the date when Romania confirmed its first positive COVID-19 case in 2020. Furthermore, all restrictions imposed by authorities were lifted on 8 March 2022. By dividing the study into three equally timed segments, this research aims to provide a detailed understanding of how the pandemic influenced the surgical treatment of inguinal hernias.

For this study, several inclusion criteria were established. Specifically, only patients who underwent traditional (open) surgical treatment for inguinal hernia within the six-year study period at our clinic were included, as laparoscopic and robotic approaches were not utilized in our clinic. To minimize COVID-19 transmission risk during the pandemic, all patients presenting for inguinal hernia surgery underwent SARS-CoV-2 screening with RT-PCR testing using nasopharyngeal swabs. Patients also had the option to present a negative RT-PCR test result conducted within 24 h prior to admission; however, this was feasible for only a limited number of patients due to the high costs associated with testing. RT-PCR remained the sole testing method utilized and considered valid for SARS-CoV-2 detection in the clinic; rapid tests were not accepted as part of the diagnostic protocol.

Patients awaiting RT-PCR results were isolated in designated rooms, reducing the clinic’s overall inpatient capacity, with four rooms specifically allocated for pre-test isolation. This setup significantly limited the number of admissions during the pandemic. Following a negative test result, patients were transferred to standard wards for preoperative preparations. In cases with positive results, patient management varied by infection severity: those with moderate to severe symptoms were redirected to specialized COVID-19 care facilities, while asymptomatic or mildly symptomatic patients were instructed to self-isolate for 14 days, adhering to health authority guidelines.

When all COVID-19 restrictions were lifted on 8 March 2022, the clinic resumed full operations, restoring regular ward use for all patients.

Once inclusion criteria were met, a comprehensive analysis was conducted on various patient parameters, including gender, age, and geographic background. The type of surgical intervention (elective or emergency) and the presence of pain at admission were also recorded. Clinical characteristics of the hernias were evaluated, focusing on their classification (reducible, incarcerated, or strangulated). Additionally, recurrence status and the bilateral presentation of hernias were documented.

The assessment of patient comorbidities was conducted using the Charlson Comorbidity Index (CCI) [10]. Regarding the surgical techniques employed, the Lichtenstein procedure, which incorporates the use of a synthetic mesh, was taken into consideration. In addition, traditional tissue-based techniques, such as the Bassini and Shouldice or McVay methods, were applied, which utilize autologous tissue repair without a mesh. Some surgical cases also involved concurrent procedures alongside hernia repair, including appendectomy, mesenteric novocainization, or even bowel resections, and their frequency was separately analyzed. The analysis extended to procedural metrics, including the duration of the surgical intervention, total hospitalization time, as well as preoperative and postoperative lengths of hospital stays.

For the statistical analysis and to generate the results, we utilized IBM SPSS Statistics 25 software for Windows (IBM, Armonk, NY, USA). To determine whether the numerical data followed a normal distribution, the Shapiro–Wilk test was applied, with a result of *p* < 0.05 indicating a normal distribution. Descriptive statistics were used for numerical variables, including the calculation of measures of central tendency and dispersion. For categorical variables, frequency distributions and percentages were employed to demonstrate variations across the different study periods.

To compare two independent samples, a Student’s *t*-test was utilized. When comparing more than two groups, an ANOVA (analysis of variance) test was applied to assess variance across multiple groups simultaneously. Additionally, chi-square tests were used to examine the relationships between categorical variables and identify differences in proportions.

For all statistical analyses, a *p*-value of less than 0.05 was considered statistically significant, indicating that the observed results were unlikely to have occurred by chance.

## 3. Results

In this study, we examined data from patients who received surgical treatment for inguinal hernias. Specifically, we focused on those treated during the pandemic period from 26 February 2020 to 25 February 2022. The results obtained from this period were then compared with data from both the pre-pandemic and post-pandemic phases.

### 3.1. Patient Demographics

The study involved an analysis of data collected from 604 patients with inguinal hernias who underwent surgical intervention at the First Surgery Clinic of the “Pius Brinzeu” Clinical Emergency Hospital in Timişoara. Throughout the pandemic phase, which spanned from 26 February 2020 to 25 February 2022, a total of 128 surgical procedures were conducted, accounting for 21.19% of the total. In contrast, during the pre-pandemic timeframe, there were 204 surgeries, representing 33.77%, while the post-pandemic period saw an increase to 272 surgeries, making up 45.03%. Statistical analysis using the chi-square test revealed a significant difference in the proportion of surgeries performed across the three periods, yielding a *p*-value of less than 0.001.

The average age, gender distribution, and patients’ environment of origin are presented in Table 1.

### 3.2. Key Information

Out of the 604 patients included in the study, 94 underwent emergency surgery, while 510 underwent elective surgery. The distribution of these surgery types across the three periods is presented in Table 2.

The comorbidities of the patients were analyzed using the Charlson Comorbidity Index. Over the three study periods, 265 patients had at least one associated condition. In the first period, 92 patients (45.5%) had a Charlson score of at least 1, compared to 47 patients (36.7%) during the pandemic period and 126 patients (46.3%) in the post-pandemic period. Analyzing the variation in proportions between these two periods, a *p*-value of 0.170 was obtained.

An interesting finding emerged when examining the variation in the Charlson Index for patients undergoing elective surgeries. A significantly higher proportion of patients without comorbidities were observed during the pandemic (*p* = 0.025), with 69 patients (69%) during the pandemic compared to 87 patients (53%) pre-pandemic and 132 patients (54.1%) post-pandemic.

Pain at presentation was also analyzed. Over the six-year period, 166 patients reported pain at the time of their hospital visit. In the first period, 52 patients (25.5%) presented with painful symptoms; this number increased to 37 patients (28.9%) during the pandemic and, later, to 77 patients (28.3%) in the post-pandemic period. Statistical tests presented a *p*-value of 0.730.

Clinically, patients presented with reducible, incarcerated, or strangulated hernias. The variation in these presentations over the three periods is presented in Table 3.

One of the long-term complications of inguinal hernia is recurrence. In the first period of the study, 10 patients (4.9%) who experienced a recurrence underwent surgical intervention for at least a second time. During the pandemic, 11 patients (8.6%) presented with recurrence, while in the post-pandemic period, 16 patients (5.9%) required surgical intervention for the same condition. After performing statistical tests to analyze the differences in proportions among the three groups, a *p*-value of 0.384 was obtained.

When analyzing the presence of patients who presented with bilateral inguinal hernias for surgical treatment, 4 cases (2%) were reported in the first period of the study, 10 cases (3.7%) in the post-pandemic period, and 4 cases (3.1%) during the pandemic period. After assessing the difference in proportions of bilateral hernia cases across the three periods, a *p*-value of 0.549 was obtained, indicating no statistically significant differences.

One of the interesting aspects investigated was the identification of patients with a history of previous inguinal hernia surgery on the opposite side. Throughout the three study periods, six patients in each period required a new surgical intervention for an inguinal hernia, but this time on the opposite side (2.9% vs. 4.7% vs. 2.2%). Statistical tests yielded a *p*-value of 0.395.

Modern hernia repair techniques increasingly involve the use of mesh. Although this method incurs higher costs, it has become a more commonly adopted procedure in our clinic. In the first period of the study, only 6 patients (2.9%) underwent repair with the use of mesh, a number that increased to 8 cases (6.3%) during the pandemic and further increased to 47 cases (17.3%) in the post-pandemic period. Statistical analysis of the differences in proportions across the three periods yielded a *p*-value of <0.001.

The types of surgical interventions applied for the treatment of inguinal hernias and the rate of postoperative complications are described in Table 4.

In this study, there was one postoperative death, which occurred during the pandemic period. Scrotal edema was reported as a complication in three cases (1.5%) during the pre-pandemic period and in two cases during the post-pandemic period, with all five cases responding favorably to conservative treatment. Additionally, postoperative scrotal hematoma was observed in two cases (1%) during the pre-pandemic period and in one case (0.4%) during the post-pandemic period.

During the pandemic, three patients (2.3%) developed postoperative SARS-CoV-2 infections, all of which were mild forms and did not negatively affect postoperative recovery. Among the patients operated on in the post-pandemic period, five (1.8%) had a history of previous SARS-CoV-2 infection. We also encountered five patients who tested positive for COVID-19 upon presentation. Consequently, their data were excluded from the study because, following the RT-PCR test results, they were either transferred to a specialized COVID-19 clinic or instructed to self-isolate at home for 14 days, in the case of asymptomatic infection.

### 3.3. Hospital Stay

The length of hospital stay across the three periods was also analyzed. The variation in hospitalization duration and the length of surgical procedures over the three periods are presented in Table 5.

## 4. Discussion

Throughout the COVID-19 pandemic, healthcare personnel, including surgeons, were redeployed to handle the overwhelming surge in patient numbers. This redistribution involved hospitals significantly reducing elective surgeries and limiting outpatient admissions due to the scarcity of hospital beds and equipment, which were fully utilized for the treatment of COVID-19 patients. The World Health Organization (WHO) reported a global mortality rate for COVID-19 of around 3% [11]. However, mortality rates are notably higher among older individuals, patients with hypertension or weakened immune systems, those undergoing surgical procedures, and cancer patients [12,13,14]. Significant reductions in the number of surgical interventions were reported globally, especially during the most disruptive 12 weeks of the COVID-19 pandemic, with estimates suggesting a decrease in elective surgeries ranging between 72.3% and 74.4% worldwide [3,15,16]. A Finnish registry study noted an overall 8% increase in waiting times for elective procedures in 2020 compared to 2017–2019, with monthly waiting times increasing by 7% to 34% from May to November [17]. Similarly, a study on elective interventions in Maryland during the pandemic reported a 55.8% reduction in procedures [18], while an Italian study found a 64.8% decrease in surgical volume during the first wave [19]. In Australia, private hospitals reported a 22.6% reduction in elective urological procedures in April 2020 compared to the previous year [20]. Meanwhile, Brazil experienced a much more dramatic decline, with a reduction of 86.9% in urological procedures [21].

Consistent with these global trends, our study revealed a significant decrease (*p* < 0.001) in the number of surgeries performed for inguinal hernia treatment, reporting a 37.2% decline compared to the pre-pandemic period. Similarly, a national study in Romania reported an initial 44.4% reduction in surgeries during the early phase of the pandemic, tapering to a 29.7% decline by the end of 2021 [16].

A comparable finding was seen in a study conducted at a university hospital in Florence, Italy, where hernia repair surgeries were reduced by 48% when comparing March 2019 to March 2020 [22]. This was a finding reported in Brazil as well, where there was a 55.21% decline in the number of IHRs performed during the pandemic [23]. In contrast, no statistically significant differences were found in age, environment of origin, or the time between admission and surgery. This outcome aligns with the findings of Gomez et al., who reported a 21% reduction in elective inguinal hernia repairs and a 17% decrease in emergency department visits for inguinal hernias [24].

A significant increase (*p* = 0.004) in the proportion of patients undergoing emergency surgeries was observed, largely attributed to the postponement of elective surgeries and to governmental restrictions and recommendations that advised people to visit hospitals solely for emergencies. Furthermore, patients’ fears of exposure to the hospital environment due to the risk of contracting the novel coronavirus contributed to a marked decrease in hospital presentations [25,26]. The analysis of “watchful waiting” in ventral hernias reported that 4% of patients required emergency surgery within a five-year period [27]. Similarly, a study by Martin et al. indicated this rate to be 8.3% [28]. In a study comparing hernia repair rates before and after the pandemic, Kollatos et al. reported an increase in emergency IHRs, accompanied by a decrease in scheduled procedures [29]. Lima et al. initially anticipated a similar trend; however, they found that the number of emergency cases was lower during the pandemic [30]. A single-center study from New York City observed a significant reduction in emergency IHR cases [17]. Conversely, increased emergency IHR rates were documented in single-center studies conducted in Turkey [31] and the United Kingdom [32]. These discrepancies in reported volumes may be attributed to regional variations in epidemiology [8].

Physical strain has been shown to significantly elevate intra-abdominal pressure, a critical factor in the onset of inguinal hernias. This increase typically occurs during activities requiring heavy lifting or intense exertion. As the abdominal wall experiences excessive tension, the likelihood of hernia formation rises, especially in individuals with inherent weaknesses in their musculature [33,34,35]. During the COVID-19 pandemic, lockdown measures significantly restricted physical and sports activities, with gyms closed and outdoor exercise limited. Inguinal hernias, often caused by physical exertion, such as heavy lifting or high-impact sports, may have seen a decline in incidence due to this reduced physical activity. Due to the restrictions imposed during the pandemic, many individuals adopted more sedentary lifestyles, which may have contributed to a decrease in the incidence of hernias and a reduction in hospital visits and hernia diagnoses during lockdown periods. This trend aligns with the overall reduction in physical exertion and activity levels during this time [16,36].

In our study, we observed a decrease in the proportion of male patients during the pandemic, a trend that continued into the post-pandemic period. The literature presents varying perspectives on this issue. Some studies report a significant increase in the proportion of female patients during the pandemic [7,31]. Conversely, other researchers do not indicate a notable shift in gender distribution during this period [6,36,37].

An interesting aspect of this study is the variation in the Charlson Comorbidity Index among patients undergoing elective surgery. A significantly higher proportion of patients without comorbidities was observed during the pandemic. It is well established that patients with comorbidities faced an increased risk of severe outcomes in the event of a SARS-CoV-2 infection. Consequently, patients with multiple comorbidities who had non-emergent hernias (i.e., non-incarcerated or non-strangulated) opted to postpone surgical treatment during the pandemic, leading to a higher proportion of patients without comorbidities undergoing surgery. These individuals were considered less susceptible to severe COVID-19 outcomes in the event of infection and had less fear of undergoing surgical treatment. This observation is supported by literature, which reports a significant decrease in the number of patients with multiple comorbidities undergoing surgery during the pandemic [38]. However, there are also studies that report similar proportions of comorbid patients across both periods [28].

Regarding the characteristics of patients’ hernias, the proportions of reducible, incarcerated, or strangulated hernias did not vary significantly across the three periods. However, a slight increase of approximately 5% in reducible hernias was observed during the post-pandemic period. This may be attributed to increased awareness of the risks associated with incarceration or strangulation [39,40,41], leading patients to opt for surgical treatment earlier in order to avoid additional postoperative complications [42]. In a Romanian national study, a decrease in the number of patients with both reducible (36%) and incarcerated/strangulated hernias (47%) was observed during the first phase of the pandemic, though the proportion of these cases did not show a statistically significant variation between the pre-pandemic and post-pandemic periods [16].

Although no significant global variation in the type of surgical procedures performed has been reported [7,16,28,43], in our clinic, the post-pandemic period saw an increase in the use of the Lichtenstein procedure, which involves the use of mesh. The effectiveness of mesh in preventing recurrence following surgical hernia repair has been well established [44]. Once the pandemic subsided and healthcare resources were more equitably redistributed [7,8,16], mesh usage in these patients became more frequent [45]. It is also worth noting the occurrence of three cases of Amyand’s hernia [46], none of which were reported during the pandemic period.

Regarding postoperative complications, their proportion did not vary significantly across the three periods. In general, the incidence of complications in hernia surgery remained consistent, as reported in the literature. Kudsi et al. [36] noted the absence of significant changes in complication rates, a finding corroborated by Turan et al. [31], who demonstrated that differences in the proportion of complications, according to the Clavien–Dindo Classification, did not significantly vary between the pre-pandemic and post-pandemic periods.

One of the primary objectives of the medical team in our clinic during the pandemic was to minimize the risk of exposing patients to the novel coronavirus. Consequently, a significant reduction in both postoperative hospitalization duration and total hospital stay was observed in the clinic where the study was conducted. However, there was no notable change in preoperative hospitalization duration compared to the pre-pandemic period. This can be attributed to the clinic’s management protocols during the pandemic, which required patients to be admitted only after undergoing RT-PCR testing for COVID-19 detection and being isolated for 24 h or until the test results were available. If the test results were negative, the patients underwent surgery the following day. Among the elective surgery patients, five tested positive upon admission. These patients opted for home isolation for 14 days as per the recommended guidelines.

Regarding emergency admissions, these patients were also tested for COVID-19 upon presentation to the emergency department, and none tested positive. Beyond these measures, the goal of the surgeons was to limit the patients’ exposure to the hospital environment and staff, ensuring a reduced length of hospital stay. Patients were discharged once they presented a good general condition, intestinal transit was present, hemodynamic stability was achieved, and no postoperative complications were observed. This strategy has been reported globally for the surgical management of various pathologies, including pulmonary conditions and inguinal hernia cases. For example, Katzen et al., [8] reported a reduction in the average length of hospital stay for inguinal hernia surgery from 4 to 3 days.

Beyond the evident changes in the treatment of inguinal hernias during the pandemic compared to the pre-pandemic period, a key strength of this study is the presentation of the post-pandemic situation, for which the current literature offers limited relevant information. The question arises: have things returned to normal? While there has been a significant increase in the number of cases in the post-pandemic period, alongside a decrease in the proportion of emergency surgeries, there is a clear shift in patient mentality. Patients now seek medical services more promptly, choosing not to delay treatment, and avoid waiting until their hernia becomes incarcerated or strangulated.

It is undeniable that healthcare systems worldwide were supported to manage the pandemic and the influx of infected patients, necessitating reorganization and resource redistribution. As a result, there has been an increase in the number of patients undergoing Lichtenstein surgical treatment in our clinic. Additionally, the length of hospital stays has decreased significantly in the post-pandemic period compared to both the pre-pandemic and pandemic phases. Surgeons have become more efficient in managing postoperative care, allowing for a higher turnover of patients and providing surgical treatment to more individuals, ultimately aiming to reduce the need for emergency surgeries.

This study demonstrates that even in times of crisis, when specific protocols are followed, inguinal hernia treatment can be performed safely without catastrophic outcomes for patients. It also underscores that delaying treatment is not a viable solution, as timely surgical intervention ensures better patient outcomes.

### Study Limitations

Beyond the strength of this study, which lies in the presentation of the post-pandemic situation, it is important to note that this is a single-center, retrospective study conducted within one surgical clinic, and the results should be analyzed and interpreted with caution. Although a comprehensive national study conducted over a three-year period that covers various aspects of inguinal hernia treatment and beyond already exists [1], there is a need for larger studies designed to examine the dynamics of both the pandemic and post-pandemic periods to better understand the medium- and long-term impacts of one of the most significant global crises in human history.

Another limitation of this study is the exclusive use of traditional (open) hernia treatment methods in our clinic, without incorporating laparoscopic or robotic approaches. Patients undergoing these more advanced techniques may experience different postoperative outcomes, with shorter recovery times and hospital stays. Additionally, this cohort of over 600 patients was operated on by multiple surgeons, and their individual preferences for surgical techniques must also be taken into account. Lastly, it is essential to consider the patients who had inguinal hernias but succumbed to COVID-19 during the pandemic without ever presenting to the hospital. The absence of data on these individuals represents a significant limitation to this study. The true impact of delayed or foregone surgical intervention in such cases remains unknown, and the inability to account for these outcomes limits the comprehensiveness of the analysis. Future research should aim to include data on patients who were unable to seek medical care due to the pandemic, as this would provide a more complete understanding of the pandemic’s effects on hernia-related morbidity and mortality.

Despite these limitations, the study demonstrates that conditions appear to be returning to normal, as evidenced by the increasing number of reducible hernias in recent periods, indicating that patients are seeking medical attention earlier. Moreover, it highlights that even in times of crisis, when epidemiological rules and principles are followed, surgical treatment can be safely performed without the need for delays, thereby avoiding the risks associated with postponing essential medical care.

## 5. Conclusions

This study highlights the significant impact that the COVID-19 pandemic had on inguinal hernia repair surgeries, particularly in terms of the shift from elective to emergency procedures. The postponement of elective surgeries, necessitated by the reallocation of healthcare resources to manage the pandemic, resulted in a notable increase in emergency surgeries and prolonged surgical durations during the pandemic period. Despite these challenges, postoperative complications remained minimal across the three study periods, demonstrating the resilience of the surgical teams and their ability to adapt to crisis conditions.

The post-pandemic period saw a recovery in elective surgery volumes and a significant increase in the use of mesh repairs, reflecting a return to pre-pandemic surgical practices with an added emphasis on improving patient outcomes. This shift also underscores the importance of timely intervention to prevent complications associated with untreated hernias, such as incarceration and strangulation, which can lead to more complex and riskier emergency surgeries.

The findings of this study emphasize the need for healthcare systems to develop resilient strategies that can ensure the continuity of elective surgical care during global health crises. The ability to balance emergency and elective procedures is crucial in minimizing the long-term effects of delayed surgeries on patient outcomes.

## Figures and Tables

**Table 1:** Demographic aspects of the cohorts.

Variables	Pre-Pandemic	Pandemic	Post-Pandemic	*p*
	*n*= 204	*n* = 128	*n* = 272	
**Gender**				
**Male**	201 (98.5%)	116 (90.6%)	242 (89%)	<0.001
**Female**	3 (1.5%)	12 (9.4%)	30 (11%)	
**Age**	57.14 ± 16.61	58.38 ± 15.2	59.43 ± 15.85	0.302
**(years, M ± SD)**				
**Environment**				
**Urban**	128 (62.7%)	73 (57%)	154 (56.6%)	0.366
**Rural**	76 (37.3%)	55 (43%)	118 (43.4%)	

M = mean, SD = standard deviation.

**Table 2:** Elective vs. emergency surgery.

Variables	Pre-Pandemic	Pandemic	Post-Pandemic	*p*
**Surgery type**				
**Elective**	166 (81.4%)	100 (78.1%)	244 (89.7%)	0.004
**Emergency**	38 (18.6%)	28 (21.9%)	28 (10.3%)	

**Table 3:** Clinical characteristics of the hernias.

Variables	Pre-Pandemic	Pandemic	Post-Pandemic	*p*
**Reducible**	166 (81.4%)	104 (81.3%)	234 (86%)	
**Incarcerated**	36 (17.6%)	23 (18%)	38 (14%)	0.371
**Strangulated**	2 (1%)	1 (0.8%)	0	

**Table 4:** Surgical techniques and postoperative complication rates.

Variables	Pre-Pandemic	Pandemic	Post-Pandemic	*p*
**Surgical techniques**				<0.001
Autologous tissue repair	198 (97%)	120 (93.7%)	225 (82.7%)	
Lichtenstein repair	6 (2.9%)	8 (6.2%)	47 (17.2%)	
**Associated interventions**				
Appendectomy	2 (1%)	0	1 (0.4%)	
Mesenteric novocainization	6 (2.9%)	5 (3.9%)	2 (0.7%)	
Bowel resection	2 (1%)	1 (0.8%)	1 (0.4%)	
**Postoperative complications**	5 (2.5%)	1 (0.8%)	3 (1.1%)	0.368

**Table 5:** Hospital stays and lengths of surgical procedures.

Variables	Pre-Pandemic	Pandemic	Post-Pandemic	*p*
**Duration of surgery**	103.55 ± 35.39	146.85 ± 58.66	143.24 ± 50.69	<0.001
**(min., M ± SD)**				
**Preoperative hospitalization**	1.33 ± 1.24	1.43 ± 1.28	1.33 ± 1.25	0.587
**(days, M ± SD)**				
**Postoperative hospitalization**	5.06 ± 2.8	4.1 ± 2.35	3.61 ± 2.77	<0.001
**(days, M ± SD)**				
**Total hospitalization**	6.4 ± 3.08	5.53 ± 2.82	4.9 ± 3.27	<0.001
**(days, M ± SD)**				

M = mean, SD = standard deviation.

## Data Availability

The datasets used and/or analyzed during the current study are available from the corresponding author upon reasonable request.
